# Prediction of subjective cognitive decline after corpus callosum infarction by an interpretable machine learning-derived early warning strategy

**DOI:** 10.3389/fneur.2023.1123607

**Published:** 2023-06-09

**Authors:** Yawen Xu, Xu Sun, Yanqun Liu, Yuxin Huang, Meng Liang, Rui Sun, Ge Yin, Chenrui Song, Qichao Ding, Bingying Du, Xiaoying Bi

**Affiliations:** Department of Neurology, Changhai Hospital, Second Military Medical University, Shanghai, China

**Keywords:** corpus callosum infarction, cognitive impairment, machine learning, subjective cognitive decline, Shapley additive explanations

## Abstract

**Background and purpose:**

Corpus callosum (CC) infarction is an extremely rare subtype of cerebral ischemic stroke, however, the symptoms of cognitive impairment often fail to attract early attention of patients, which seriously affects the long-term prognosis, such as high mortality, personality changes, mood disorders, psychotic reactions, financial burden and so on. This study seeks to develop and validate models for early predicting the risk of subjective cognitive decline (SCD) after CC infarction by machine learning (ML) algorithms.

**Methods:**

This is a prospective study that enrolled 213 (only 3.7%) CC infarction patients from a nine-year cohort comprising 8,555 patients with acute ischemic stroke. Telephone follow-up surveys were carried out for the patients with definite diagnosis of CC infarction one-year after disease onset, and SCD was identified by Behavioral Risk Factor Surveillance System (BRFSS) questionnaire. Based on the significant features selected by the least absolute shrinkage and selection operator (LASSO), seven ML models including Extreme Gradient Boosting (XGBoost), Logistic Regression (LR), Light Gradient Boosting Machine (LightGBM), Adaptive Boosting (AdaBoost), Gaussian Naïve Bayes (GNB), Complement Naïve Bayes (CNB), and Support vector machine (SVM) were established and their predictive performances were compared by different metrics. Importantly, the SHapley Additive exPlanations (SHAP) was also utilized to examine internal behavior of the highest-performance ML classifier.

**Results:**

The Logistic Regression (LR)-model performed better than other six ML-models in SCD predictability after the CC infarction, with the area under the receiver characteristic operator curve (AUC) of 77.1% in the validation set. Using LASSO and SHAP analysis, we found that infarction subregions of CC infarction, female, 3-month modified Rankin Scale (mRS) score, age, homocysteine, location of angiostenosis, neutrophil to lymphocyte ratio, pure CC infarction, and number of angiostenosis were the top-nine significant predictors in the order of importance for the output of LR-model. Meanwhile, we identified that infarction subregion of CC, female, 3-month mRS score and pure CC infarction were the factors which independently associated with the cognitive outcome.

**Conclusion:**

Our study firstly demonstrated that the LR-model with 9 common variables has the best-performance to predict the risk of post-stroke SCD due to CC infarcton. Particularly, the combination of LR-model and SHAP-explainer could aid in achieving personalized risk prediction and be served as a decision-making tool for early intervention since its poor long-term outcome.

## 1. Introduction

The corpus callosum (CC) is the largest commissural bridge of white-matter fibers between bilateral hemispheres (1), accompanied by a unique anterior and posterior double circulation system and abundant collateral arteries (2). Because of the sufficient blood supply, CC infarction is extremely rare and accounts for barely 2.3–8.0% of cerebral ischemic stroke (3–5). Because of its unique physiological structure and function, the manifestations of CC infarction are variable and lacking of specificity. Due to these special and complex characteristics, misdiagnosis and delayed treatment are not uncommon for CC infarction (6). Interestingly, we previously found that, compared to general basal ganglia infarction, patients with CC infarction had lower National Institutes of Health Stroke Scale scores and better recovery at the time of discharge, while the one-year mortality is higher with poorer long-term prognosis (5). Cognitive impairment is one of the main causes of poor long-term prognosis in patients with CC cerebral infarction. Unfortunately, due to it’s occult exacerbation process, patients often do not pay enough attention to it in the early stage, and miss the optimal intervention period, resulting in irreversible cognitive impairment.

Subjective cognitive decline (SCD) is an individual’s self-report of cognitive decline and is nowadays thought to be a precursor to various common cognitive disorders in clinic, such as mild cognitive impairment (MCI) (7) and Alzheimer’s disease (AD) (8). Recent researches have revealed that compared with age-matched healthy controls, patients with SCD suffer a 4.5–6 times higher risk of developing into MCI or AD (9, 10). Compared to universally-known post-stroke cognitive impairment (PSCI), SCD places more emphasis on the patient’s subjective perceptions and timely feedback from caregivers, making it easier to identify and intervene early. Meanwhile, our, as well as others’ previous studies have proved that, white matter lesions (WMLs) are important pathological mechanisms for cognitive dysfunctions (5, 11–14). As an extremely rare subtype of stroke with prominent WMLs, CC infarction is likely to become a potential driver of SCD and other symptomatic cognitive decline. Therefore, aiming to restore brain health and cognitive abilities as long as possible, this at-risk group is recognized as an eligible target population for early intervention strategies (15, 16).

The role of physicians has always been to synthesize the data available to them to identify prognosis patterns that guide early intervention. Machine learning (ML) is a new rising technical foundation of artificial intelligence, which enables the computer to learn the rules hidden in the data automatically (17). Several studies have revealed that ML-based models are promising in predicting the diagnosis, prognosis or recurrence of ischemic stroke, what’s more, those models are also widely used in the field of psychology, biomechanics and so on (18–23). Nevertheless, it still lacks of ML-based evidence on SCD prediction after cerebral infarction. What’s more, the “black-box” character of ML-technique hinders clinicians to have a good understand of the predictive decision, namely failure in accountability (24). To this end, we proposed an interpretable strategy combining ML algorithm with SHapley Additive exPlanations (SHAP) to provide consistent and locally accurate attribution values for each feature within each prediction model. It’s calculated by comparing the predicting discrepancy in all possible combinations containing and withholding each feature and provide a unique report individually (25).

Here, with the largest sample of CC infarction to date, this is an exploratory study that for the first time emphasizes the clinical feasibility to individually predict the occurrence of one-year SCD after CC infarction by using ML methods. We also attempt to apply SHAP-value for explaining the importance and influence of each predictor contributing to the optimal model’s outcome. We expect this ML-derived early warning system and SHAP-based framework of interpretation could help clinicians to better counsel patients, conduct targeted follow-up and determine personalized interventional measures.

## 2. Methods

### 2.1. Participants

**Figure 1:**
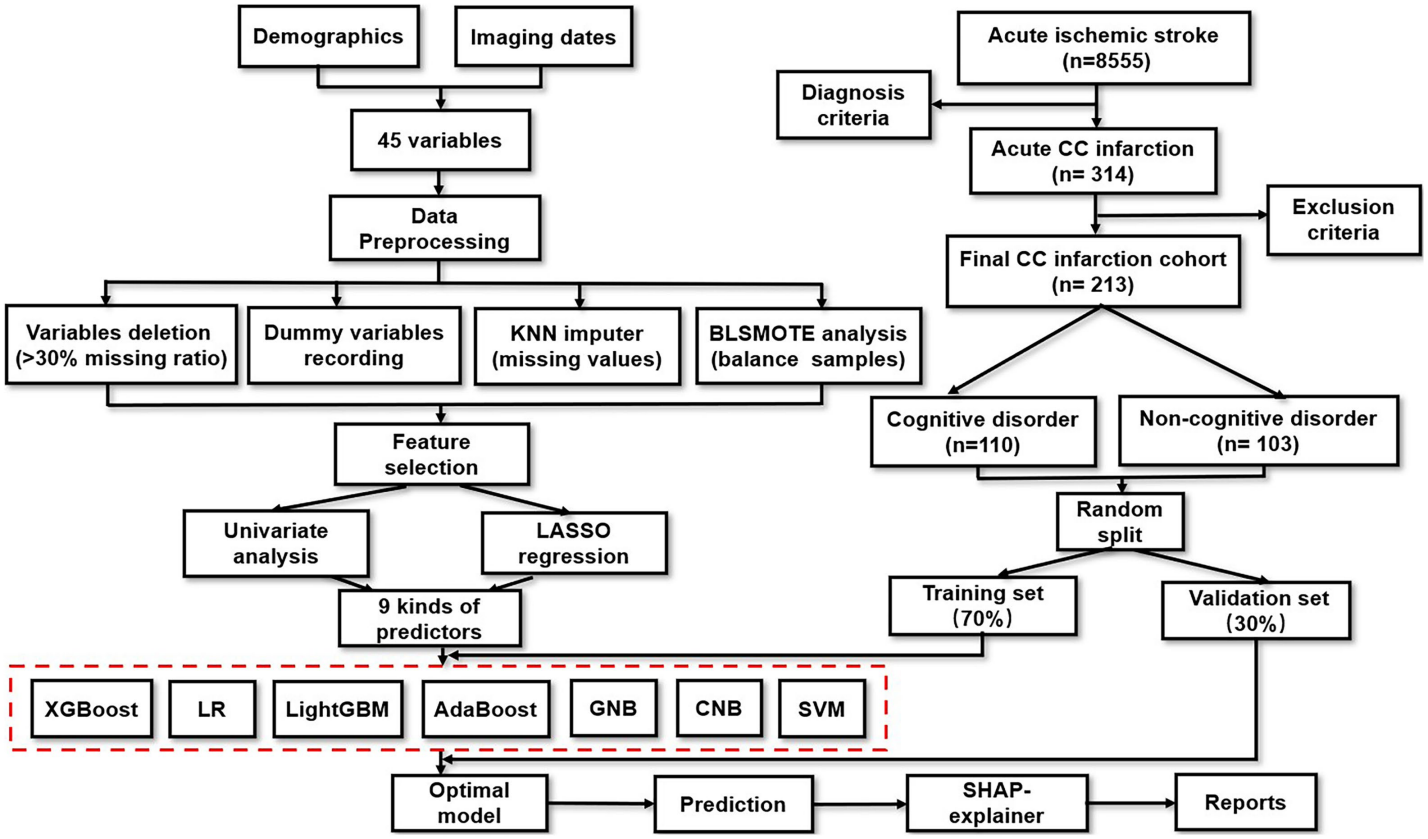
Schematic representation of the study design and modeling workflow.

The design of this study is presented in Figure 1. A total of 8,555 ischemic stroke patients were collected from Shanghai Changhai Hospital between July 2012 and June 2021. Among them, 314 (3.7%) patients with acute CC infarction were enrolled. The exclusion criteria were as follows: (i) age under 30 or above 80 years old, (ii) cognitive impairment precedes CC infarction, (iii) follow-up period was less than 1 year, or loss to follow-up, (iv) serious medical complications, (v) incomplete neuroimaging materials, (vi) acceptance of thrombolytic therapy or interventional therapy, and (vii) failure to sign written informed consent. Ultimately, 213 patients with acute CC infarction were included for final analysis. This study was approved by the Changhai Hospital Ethics Committee (NO. CHEC2021-1021).

### 2.2. Clinical and imaging assessment

Basic clinical and imaging information of enrolled patients were obtained from Electronic Medical Record (EMR) management system. A list of these variables was shown in Supplementary Table S1, including: demographic characteristics (age, sex, body mass index[BMI]), vascular risk factors (hypertension, diabetes mellitus, prior stroke or transient ischemic attack, heart diseases, smoking, alcoholism), stroke severity on admission (time from onset to hospital, NIH stroke scale [NIHSS] scores), laboratory tests (alanine transaminase [ALT], low-density lipoprotein [LDL], high-density lipoprotein [HDL], cholestenone, triglyceride, creatine, urea, uric acid, Glucose [Glu]), (thyroid-stimulating hormone [TSH], triiodothyronine [T3], thyroxine [T4], erythrocyte, leukocyte, neutrophil to lymphocyte ratio [NLR], hemoglobin, thrombocyte, erythrocyte sedimentation rate [ESR], C-reactive protein [CRP], homocysteine [Hcy], glycosylated hemoglobin [HbA1c], fibrinogen [FIB], D-dimer), imaging examination assessment (pure CC infarction, infarction subregion of CC, other infarction areas, location of angiostenosis, number of angiostenosis, extracranial carotid plague, TOAST subtype (26)), functional status (Modified Rankin scale [mRS] at 3-month), secondary prevention and recurrence (rehabilitation treatment, regular secondary prevention and recurrent stroke).

In detail, rehabilitation here referred to a series of standardized rehabilitation therapy obtained in rehabilitation hospitals, which mainly focuses on the motor and language function. Moreover, it also included lifestyle modification and taking medication exactly as prescribed at Discharge Notes, as well as additional carotid surgery or stenting, repairment for closure of patent foramen ovale, and surgery for intracranial or vertebral stenosis if necessary (27).

**Figure 2:**
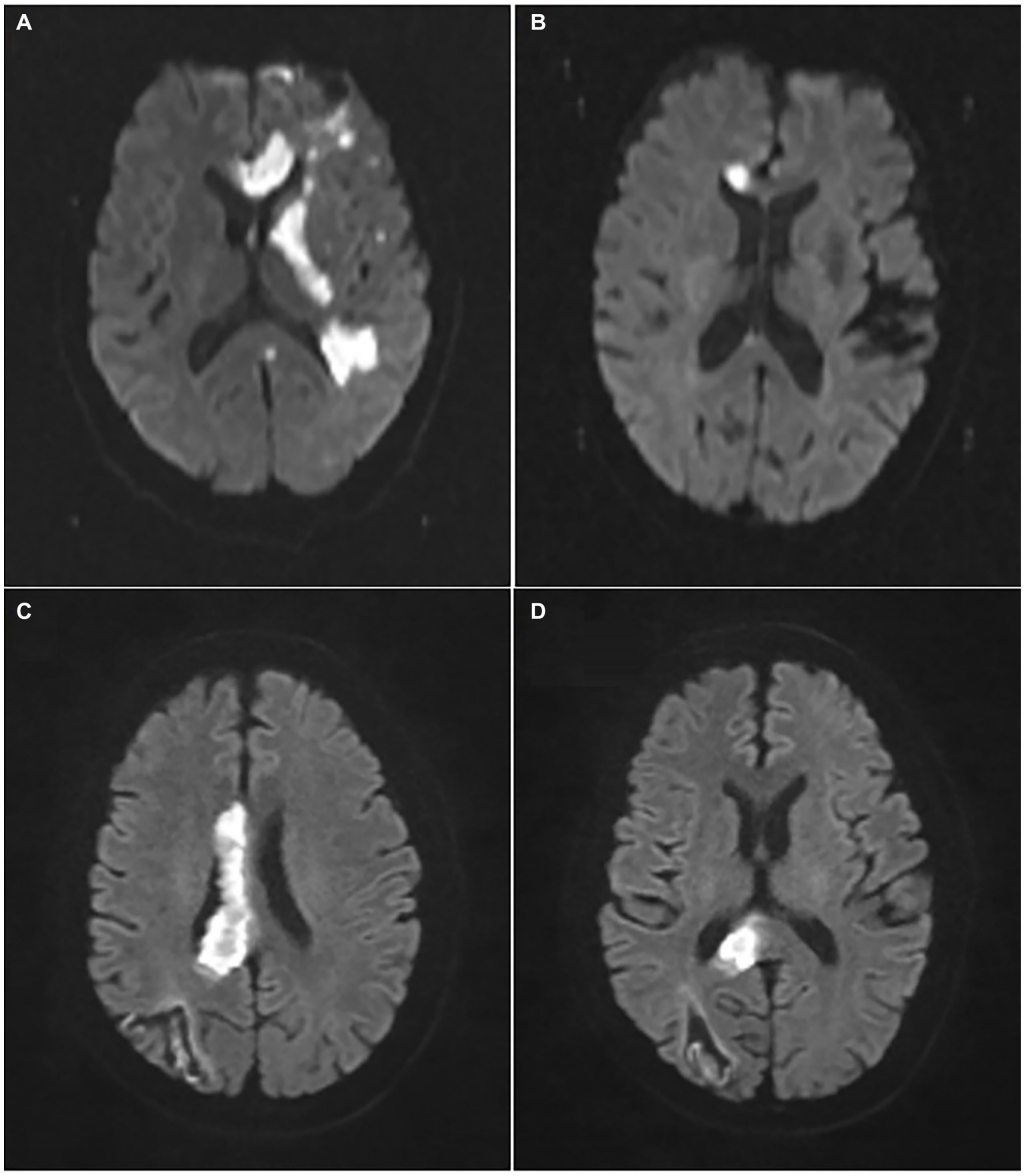
Representative images of pure and complex callosal infarction. **(A)** Complex callosal infarction. **(B)** Pure genu infarction of the corpus callosum. **(C)** Pure body infarction of the corpus callosum. **(D)** Pure splenium infarction of the corpus callosum.

Additionally, the corresponding neuroimaging evidences were collected from both (i) MRI (Magneton Impact 3.0 T, Siemens, Berlin, Germany), including T1-imaging, T2-imaging and diffusion-weighted imaging (DWI), and (ii) MR-angiography (MAGNETOM Skyra 3.0 T, Siemens) or CT-angiography (Aquillion One, Toshiba, Tokyo, Japan). As shown in Figure 2, the patients could be divided into 2 groups according to DWI patterns: pure callosal infarcts and complex callosal infarcts. The former was further subdivided into following subgroups: (i) Pure genu infarction of the corpus callosum, (ii) Pure body infarction of the corpus callosum, and (iii) Pure splenium infarction of the corpus callosum according to the subregions of CC.

### 2.3. Cognitive dysfunction definition

Telephone follow-up surveys were carried out for the patients with definite diagnosis of CC infarction one-year after onset. According to the cognitive decline module of the Behavioral Risk Factor Surveillance System (BRFSS), which is the largest ongonging health survey system in the world (28). SCD was identified by the question of BRFSS, “During the past 12 months, have you experienced confusion or memory loss that is happening more often or is getting worse?” (29–31). If respondents had a clear cognitive complaint compared with the self-perception before stroke, they were classified as suffering from post-stroke SCD, otherwise they were distinguished as non-SCD. Additionally, there were five detailed questions of aggravating confusion or memory decline mentioned in the BRFSS questionnaire, including: (1) the frequency of giving up daily household activities or common chores, (2) the frequency of requirement of assistance with these daily activities, (3) the frequency of getting help, just as you wanted, (4) the frequency of work, volunteer, or social activities disturbed by the confusion or memory disorder, and (5) whether having sought medical attention for this (29, 31). These SCD-related outcomes evaluated by a five-point scale (Always, usually, sometimes, rarely, never) were dichotomized to determine if these outcomes were challenge (assigned as 1) vs.if they rarely or never happened (assigned as 0) (28). Consequently, the patients would better realize whether they had problems with post-stroke SCD and SCD related functional impairment through our telephone survey.

### 2.4. Machine learning

#### 2.4.1. Features selection

Least absolute shrinkage and selection operator (LASSO) was used to select variables among high-dimensional data based on the penalty method. The originally small coefficients were compressed to 0 after compressing (32). Thereafter, regarded as non-significant variables, the corresponding variables of these coefficients were directly discarded (33). LASSO regression is also usually characterized by variable selection and complexity adjustment for construction of ML models while avoiding overfitting. However, the most ML methods could not process data with missing values, so we imputed the dataset by KNN before LASSO regression. In our study, this binary logistic regression (LASSO) model is helpful to screen out significant predictors of SCD after acute CC infarction.

#### 2.4.2. Machine learning models

Then, the dataset was randomly divided into training set and validation set. As in most cases, the training set accounted for 70% and the validation set accounted for 30% (34). Seven comprehensive and up-to-date ML algorithms were thereafter used to develop the predictive models, including Extreme Gradient Boosting (XGBoost), Logistic Regression (LR), Light Gradient Boosting Machine (LightGBM), Adaptive Boosting (AdaBoost), Gaussian Naïve Bayes (GNB), Complement Naïve Bayes (CNB), and Support vector machine (SVM). For each ML-based model, five-fold cross-validation was performed to evaluate the generalization ability (35), and the optimal hyperparameters were selected subsequently. Additionally, the following indicators are calculated to comprehensively evaluate the performance of different models: area under the curve (AUC)-value, accuracy, sensitivity, specificity, positive predictive value (PPV), negative predictive value (NPV), and F1 scores.

#### 2.4.3. Personalized interpretation

Specifically, we further utilized a novel approach to explain the output of the highest-performing ML model, namely Shapley Additive explanation (SHAP), rooted in Shapley value. Calculated the marginal contribution of a feature when it is added to the “black-box” model, then the SHAP value takes the average value considering the different marginal contribution of the feature in all permutations of individuals (36). A feature with a positive SHAP value improves the output value, and those larger numerical values make greater contributions (37). In our study, the SHAP summary plot, the importance ranking, and the SHAP dependence plot of the relevant covariates were used to improve the interpretability. SHAP explainer was suitable to visualize the black-box ML algorithms on the basis of the cooperative game theory (36). The advantage of SHAP method is be able to explain how much and in which direction each predictor influences the optimal ML-model’s output. It concluded that, the core idea of SHAP-explainer is to calculate the marginal contribution of features to model output, and then to explain the “black-box” model from global and local levels (38).

### 2.5. Data preprocessing

Firstly, indicators including ESR, CRP, TSH, T3, T4 were excluded because of the high missing ratio (over 30% (39), respectively). Secondly, categorical variables were encoded into dummy variables, and the details were as follows: (i) TOAST subtypes were converted into range 1–5 (LAA = 1, CE = 2, SAO = 3, ODC = 4, UND = 5), (ii) Infarction region of CC were divided into range 1–5 (rostrum = 1, genu = 2, body = 3, splenium = 4, at least two of rostrum, genu, body and splenium = 5), and (iii) Other infarction areas were turned into range 0–5 (none = 0, frontal lobe = 1, parietal lobe = 2, temporal lobe = 3, occipital lobe = 4, others = 5), (iv) Location of angiostenosis were encoded into range 0–4 (none = 0, ICA = 1, VBA = 2, both of ICA and VBA = 3), etc. After that, remaining indicators were processed by K-nearest-neighbor (KNN) analysis to impute their missing values (40). In the end, the Borderline-1 SMOTE (BLSMOTE) algorithm was also adopted to balance the samples between the SCD group and non-SCD group in an absolute fairness (for 50%, respectively), which would improve the reliability or classifying performance of the ML-models (41).

### 2.6. Statistical analysis

Continuous data were uniformly described as mean (SD) or median (IQR), while categorical data were presented as n (%). Baseline characteristics were compared between the SCD group and non-SCD group after CC infarction by Chi-square test (categorical variables), two-sample *t*-test (continuous variables with symmetric distribution), Mann–Whitney *U* test (continuous variables with asymmetric distribution), or Welch’s *t*-test (continuous variables with heterogeneity of variance), as appropriate. Then, variables with a relatively remarkable (*p* < 0.1) association with cognitive outcome in univariable analysis were further analyzed by multivariable analysis with a traditional forward stepwise selection. All statistical analyzes were performed using programming language R package (version 3.6.3, https://cran.r-project.org/bin/windows/base/) and all ML-relevant workflows were performed using python (version 3.7, https://www.python.org/getit/); *p* < 0.05 indicates statistical significance.

## 3. Results

### 3.1. Demographics

The baseline demographical, clinical, biochemical and neuroimaging characteristics of 213 patients (75 female) with acute CC infarction were summarized in Supplementary Table S1. The average age at baseline was 63 [55, 69] years. After 1 year follow-up period, 110 subjects developed into post-stroke SCD, while the remaining were no-complaint (NC) patients. Compared to NC participants, SCD patients tended to be slightly older (63 [58, 71] vs. 61 [51, 68] years, *p* = 0.012), had a higher percentage of female (45.4% vs. 24.2%, *p* = 0.001), and higher mRS scores at 3 month (1 [1–3] vs. 1 [0–2], *p* <0.001). Pure CC infarction seems to be more likely to cause post-stroke SCD (*p* = 0.030). Meanwhile, the group with more than two subregions involvement of CC infarction were especially vulnerable to post-stroke SCD (*p* = 0.001). Furthermore, patients with post-stroke SCD were prone to have multiple angiostenosis with both of internal carotid artery (ICA) and vertebral basilar artery (VBA) involved (*p* = 0.009).

### 3.2. Multivariable analysis of risk factors

According to traditional forward selection, we found that female (OR: 3.344; 95% CI: 1.656–6.998; *p* = 0.001), 3-month mRS scores (OR: 1.380; 95% CI: 1.109–1.736; *p* = 0.005), pure CC infarction (OR: 4.823; 95% CI: 1.531–17.919; *p* = 0.011) were the eligible independent risk factors for SCD after acute CC infarction (Table 1). Compared with the patients with acute infarction of the genu, patients with infarction of the splenium but not rostrum or body were more likely to have cognitive deterioration during follow-up (OR: 3.058; 95% CI: 1.221–8.183; *p* = 0.020). Furthermore, the patients with at least two subregions of CC infarction were more susceptible to post-stroke SCD than those only with lesions in the genu (OR: 7.370; 95% CI: 2.649–22.124; *p* < 0.001).

**Table 1:** Multivariate logistic regression for the risk factors of post-stroke SCD after callosal infarction.

Variables	Odds ratio (95% CI)	*p* value
Age, year	1.024 (0.995–1.055)	0.114
Female (yes vs. no)	3.344 (1.656–6.998)	0.001
Uric acid, umol/L	0.999 (0.997–1.001)	0.497
Hypertension (yes vs. no)	1.266 (0.615–2.619)	0.522
3-month mRS scores	1.380 (1.109–1.736)	0.005
Pure CC infarction (yes vs. no)	4.823 (1.531–17.919)	0.011
Number of angiostenosis		
none	1 (ref)	
Seldom	0.537 (0.169–1.662)	0.284
Multiple	1.711 (0.723–4.141)	0.225
Infarction subregion of CC		
Genu	1 (ref)	
Body	3.347 (0.747–15.754)	0.116
Splenium	3.058 (1.221–8.183)	0.020
At least two of above subregions	7.370 (2.649–22.124)	<0.001

mRS, Modified Rankin scale; CC, Corpus callosum. In this table, the term of Multiple means the number of narrowed or occluded vessels is greater than two, and the term of Seldom means the level was between none and multiple.

### 3.3. Performance of machine learning models

**Figure 3:**
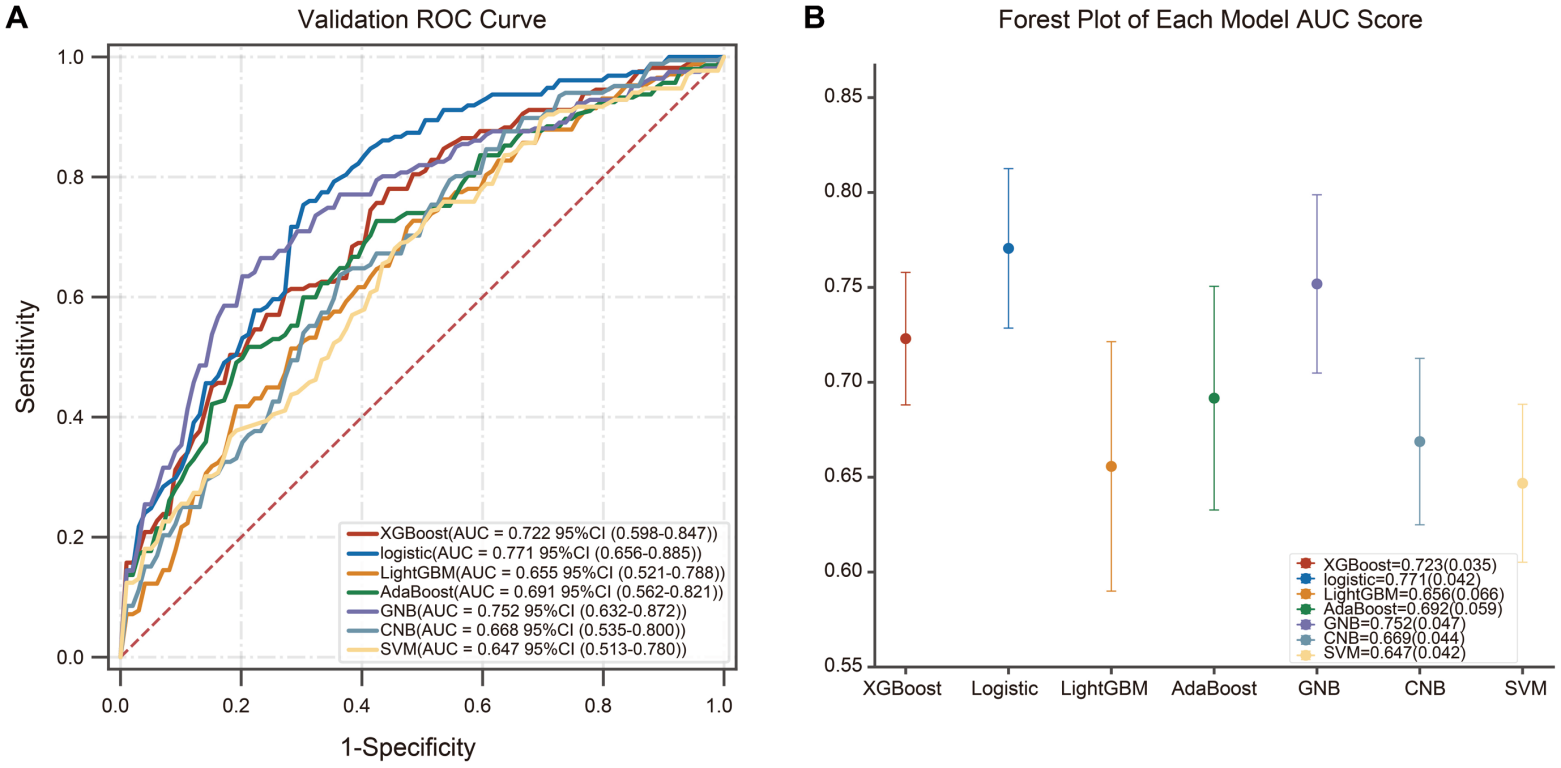
The ROC-curves and Forest map of AUC values for seven models. **(A)** The ROC curves for the different machine learning algorithms, and LR model yielded the greatest AUC among all the models. **(B)** The Forest map of AUC values of the seven models. The dots mean the AUC-value of each model, and the confidence intervals are depicted by the vertical lines.

Based on the predictors selected by LASSO in the supplementary materials (Supplementary Figure S1), different artificial intelligence (AI) -derived models were constructed (Table 2). According to the metrics, the AUC and accuracy of the LR model were obviously better than those of the other six models, respectively. Therefore, the logistic model was selected as the most prominent one for predicting SCD after acute CC infarction, which achieved an AUC of 0.771 (±0.042) and accuracy of 0.703 (±0.050) in the validation set. The ROC-curves and Forest map of AUC values for the LR and the other models were shown in Figure 3.

**Table 2:** Comparison of predictive effects of different machine learning algorithms.

Models	AUC	Accuracy	Sensitivity	Specificity	PPV	NPV	F1 score
XGBoost	0.722 (0.035)	0.618 (0.024)	0.722 (0.100)	0.717 (0.120)	0.704 (0.068)	0.578 (0.022)	0.705 (0.045)
LR	0.771 (0.042)	0.703 (0.050)	0.763 (0.094)	0.730 (0.108)	0.661 (0.077)	0.765 (0.044)	0.703 (0.064)
LightGBM	0.655 (0.066)	0.615 (0.093)	0.757 (0.145)	0.559 (0.230)	0.644 (0.104)	0.604 (0.105)	0.676 (0.042)
AdaBoost	0.691 (0.059)	0.648 (0.053)	0.669 (0.070)	0.688 (0.095)	0.628 (0.087)	0.668 (0.090)	0.642 (0.051)
GNB	0.752 (0.047)	0.700 (0.045)	0.701 (0.085)	0.768 (0.087)	0.722 (0.062)	0.683 (0.062)	0.709 (0.062)
CNB	0.668 (0.044)	0.573 (0.051)	0.711 (0.185)	0.627 (0.187)	0.616 (0.051)	0.562 (0.072)	0.641 (0.079)
SVM	0.647 (0.042)	0.594 (0.026)	0.774 (0.175)	0.496 (0.204)	0.588 (0.048)	0.609 (0.069)	0.660 (0.094)

**Figure 4:**
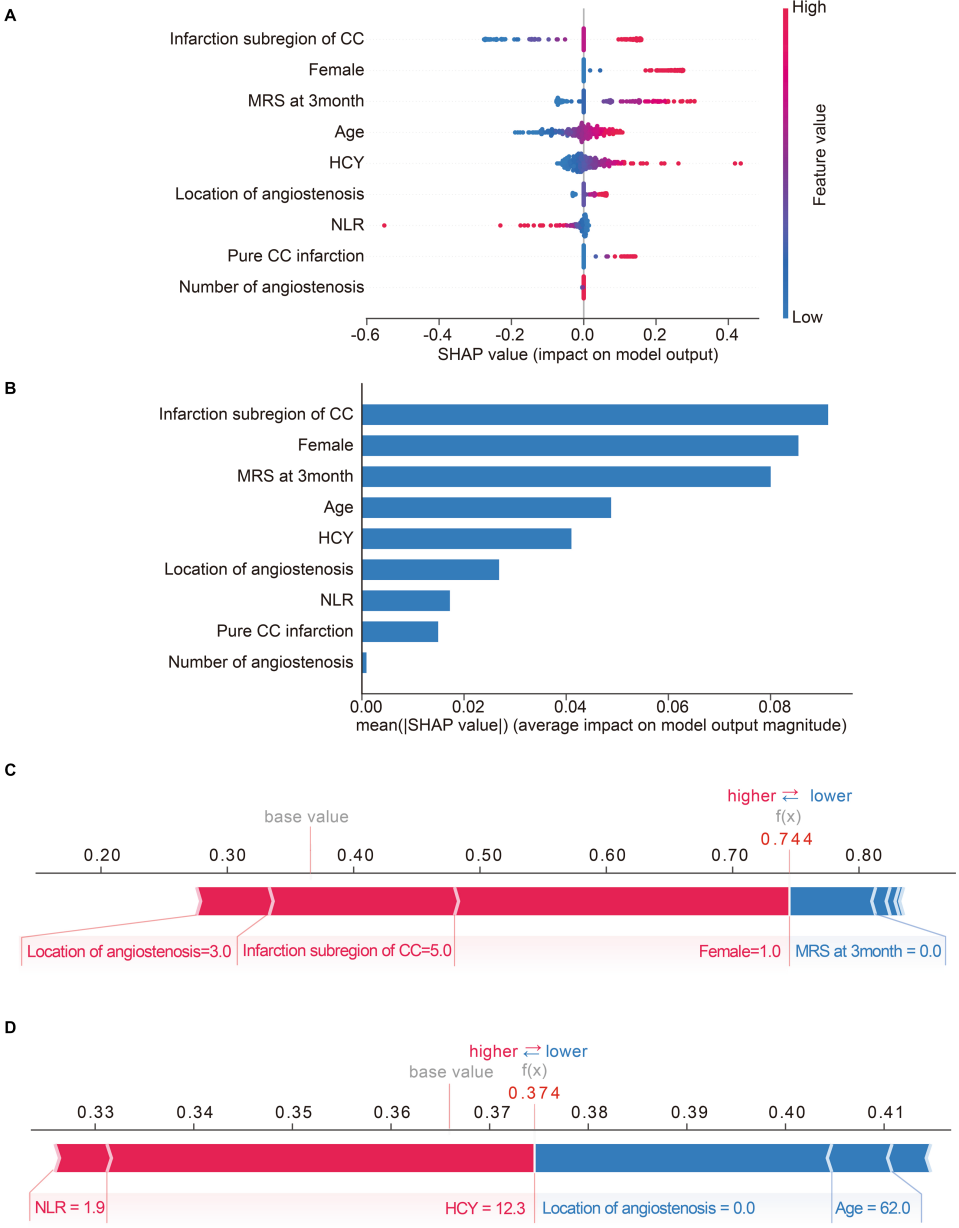
Matrix plots of the top nine important features and the SHAP plots for two selected patients. **(A)** The SHAP summary plot of LR model. Each dot represents a SHAP-value for a feature. The red color means high value, while the blue means low. The positive SHAP-value represents an increased risk of post-stroke SCD for the output of LR model, and vice versa. **(B)** The histogram of mean absolute SHA*p* values of top-nine important features of LR model. The longer the bar, the larger impact the feature has on the output. **(C,D)** SHAP force plots for two selected patients. Feature values colored red are pushing the predictive outcome towards cognitive impairment, while feature values colored blue are just the opposite. The associated Shapley value of each feature is visualized by the length of an arrow, and the longer of the arrow, the more significant the feature value is.

Moreover, we calculated the contribution of each predictor to LR model by SHAP algorithm, which can simultaneously reveal the power and direction of these factors. Thereafter, features were ranked on the basis of the absolute SHAP values over all samples (Figure 4A). As is depicted in Figure 4B, high values of infarction subregions of CC, female, 3-month mRS score and pure CC infarction have positive impact on the output of LR model, indicating the acceleration of cognitive deficit after acute CC infarction. Importantly also, age, HCY, NLR, location and number of angiostenosis were the other top-9 predictors for post-stroke SCD based on Shapely value.

SHAP model is a relatively all-powerful ML-model interpretation method, which can also be used for personalized interpretation. That means, individual patient predictions can be extracted to visualize which features played a role in their cognitive decline and what their feature values were. For instance, Figure 4C exhibits a subject with a predicted possibility of 74% for SCD after CC infarction by LR-model. The plot explains that location of angiostenosis = 3.0 (both of ICA and VBA), infarction subregion of CC = 5.0 (at least two of rostrum, genu, body, and splenium) and female = 1 (female) are the most remarkable values contributing to the increased chance of cognitive disorder, while 3-month mRS score = 0 is just the opposite. Ultimately, the result indicated a high-risk of post-stroke SCD for this subject, and the follow-up result confirmed cognitive impairment outcome, which means true positive. Similarly, Figure 4D exhibits a case with a predicted possibility of 37% for post-stroke SCD, in other words, that means a possibility of 63% for non-SCD after CC infarction. The most essential positive contributors towards adverse cognitive outcome are NLR = 1.9 and HCY = 12.3. Inversely, the negative contributors involve location of angiostenosis = 0.0 (none) and age = 62.0. Therefore, the LR-based algorithm’s result was low-risk of SCD after CC infarction for this subject, and the actual outcome was identified as non-cognitive impairment (true negative).

## 4. Discussion

The presence of SCD is known to be associated with a high risk for objective cognitive decline and even clinical progression to symptomatic disease stages (42, 43). Effective intervention to delay or prevent pathologic cognitive decline may best to targeted at the earliest symptomatice disease stage, such as SCD, in which cognitive function is still relatively preserved (44). This is an exploratory study that for the first time focuses on post-stroke SCD of rare CC infarction via an interpretable machine learning-derived early warning strategy.

After multivariate adjustment for potential confonders, we found that female, 3-month mRS scores, pure CC infarction and infarction subregion of CC independently correlated with the incidence of SCD. Interestingly, our previous study has reported that males had a higher incidence of CC infarction (5), while in the current cohort, we found females were more susceptible to SCD after this specific infarction. Reasons for this phenomenon may include: (1) females in the present study had an older onset-age of CC infarction than males (64 [58,71] vs. 62 [55,69]), (2) women are usually considered to have a lager corpus callosum volume (45–47), indicating that callosum may play a more important role in maintaining brain function of females, (3) women tend to have higher cortisol but lower estradiol levels in menopausal period (48). Indeed, scholars have well-clarified that higher serum cortisol is correlated with more severe microstructural WMLs, particularly in CC, while estrogen are thought to promote the remyelination, and the latter in turn is strongly associated with general cognitive capacity (49–51). Meanwhile, a strong interaction between serum cortisol and cerebral atrophy among females, but not males was also identified (52). Richa et al. (53) once reported that the MoCA scores (between 4–8 weeks post-infarct) were obviously correlated to mRS scores (at the same follow-up points) among the stroke patients. Then, our results showed that 3-month mRS scores were related to longer-time cognitive outcome due to CC infarction.

The structure of CC can be divided into four classical parts in the order from front to back: rostrum, genu, body and splenium (54). Consistent with previous reports (5), we found that the incidence of ‘pure’ CC infarction was rare, while the mental disturbance and cognitive dysfunction were more prominent than ‘complex’ CC infarction. The mechanisms of the discrepancy are still unclear, perhaps the atypical symptoms and insufficient distinguishment of MRI scan made it difficult to draw sufficient attention and appropriate prevention of ‘pure’ CC infarction. Meanwhile, we reported for the first time that acute infarction in the splenium had a higher tendency of cognitive decline than that in the genu. As the most vulnerable area of the CC infarction, the splenium is more vulnerable to insufficient blood supply, and the splenium lesions were known to be related with cognitive disorder, aphasia, homonymous hemianopsia, alien hand syndrome and so on (54). Therefore, we believe that the splenium plays an important role in the high incidence of SCD caused by CC infarction to some extent. What’s more, patients with at least two subregions of CC infarction were more susceptible to SCD than those only had lesions in the genu. This result is well understood given that the more structural damage CC is, the more disrupted the fiber connections and information transmission between the bilateral hemispheres. Besides, evidence showing that the infarction in body or splenium of CC could lead to disturbed executive capacity, attention and calculation (55), which may provide a side note for our viewpoint.

Besides multivariable analysis, LASSO analysis was also adopted to select potential risk predictors by eliminating irrelevant features. It is universally accepted that age was a risk factor of cognition damage after various types of ischemic stroke (56). Except of age, evidence linking high HCY(HHCY) and cognitive decline is profuse (57). It is known that, HHCY is not only associated with WMLs, but also the progression of WMLs (58). In the meantime, extensive intracranial vascular stenosis is another promotor for SCD after CC infarction. Cerebral angiostenosis/occlusion has already been proved to induce hypoperfusion and impaired executive dysfunctions, such as working memory, attention, cognitive flexibility, planning, thought organization and implementation (59). This phenomenon indicates that appropriate increase of cerebral blood flow may help prevent post-stroke SCD. Interestingly, NLR is often known as a risk factor for PSCI (60). However, we found that NLR is negatively associated with self-report cognitive decline, indicating that NLR is likely to act as a compensatory neuroprotective response in the early stage of CC infarction. Biological mechanisms between NLR and risk of post-stroke SCD have not been explored before and warrants further clarifications, especially in the condition of CC infarction.

In our study, the combination of LASSO regression and ML-based models was beneficial to identify the optimal configuration to predict whether it is vulnerable to develop SCD after CC infarction or not. Then, the seven ML algorithms were assessed by several metrics, comprising AUC-value, accuracy, sensitivity, specificity, positive predictive value (PPV), negative predictive value (NPV), as well as F1 scores. Apart from global explanation, the well-accepted local explanation, SHAP was also implemented to interpret how a complex black-box ML model makes a prediction (61). By incorporating the individualized patient profile, the level of contribution and directionality of specific input features were visualized (62). As shown in the **Table** **1**, the LR model seemed to be the best-performance classifier with the highest scores of AUC- value (77.1%), accuracy (70.3%), sensitivity (76.3%) and NPV (76.5%). In addition, acceptable values of specificity, PPV and F1 score (all above 65.0%) were achieved in the validation set. Taken together, we selected the LR model as the optimal algorithm with the best generalization ability. At the same time, we suggested that we should treat this problem dialectically and choose appropriate predictive classifier according to different clinical needs.

The strength of our research is that the cohort has the largest sample of CC infarction in the world, and the datasets are non-synthetic, which is more likely to be objective and effective as a screening tool. Unlike studies focused on each risk factor individually or its pathophysiological interpretation (63, 64), we aimed to encompass a large combination of variables from real-world clinical situations once. The variables we used, including demographics, laboratory and radiological findings were all easily accessible for clinicians, which could assist with the early prediction and prevention for suspected post-stroke SCD. Additionally, an interpretable and explainable ML model was created with the help of SHAP-explainer, promoting to make individualized clinical decisions.

There are some limitations that still needed to be ironed out in our study. Firstly, although this investigation had the maximal population of patients with acute CC infarction, the sample size was still needed to be added. Secondly, we did not exploit the different cognitive abilities separately, such as orientation, calculation, executive abilities, long-term and short-term memory and attention, etc. Thirdly, the follow-up period is not long enough to verify the proportion of patients with SCD who eventually converted to PSCI. Therefore, muti-center prospective cohorts with detailed cognitive domains impairment are needed in the future.

## 5. Conclusion

In conclusion, the present study screened out 9 key features associated with post-SCD and developed a LR-model which can improve the prediction accuracy of one-year SCD after CC infarction. What’s more, the individual report generated by SHAP facilitate the early implementation of primary prevention measures. Based on these techniques, we are even expected to continue to individually predict the long-term effects of different clinical drugs on cognitive impairment to shape a brighter future for patients with CC infarction.

## Data availability statement

The original contributions presented in the study are included in the article/Supplementary material, further inquiries can be directed to the corresponding authors.

## Ethics statement

The studies involving human participants were reviewed and approved by Changhai Hospital Ethics Committee (No. CHEC2021-1021). The patients/participants provided their written informed consent to participate in this study.

## Author contributions

YX, XS, and XB: conceived and designed the study. YX, XS, YL, YH, ML, RS, GY, CS, QD, BD, and XB: performed the study. YX, XS, YL, BD, and XB: revised the article for intellectual content. YX, XS, and BD: wrote the article. All authors contributed to the article and approved the submitted version.

## Funding

The work was financially supported by the National Natural Science Foundation of China (81871040 and 82101563), the Clinical Research Plan of SHDC (no. SHDC2020CR1038B), Scientific research project of Shanghai Health Commission (20214Y0500), and the Youth Program of Naval Medical University (2021JCQN10).

## Conflict of interest

The authors declare that the research was conducted in the absence of any commercial or financial relationships that could be construed as a potential conflict of interest.

## Publisher’s note

All claims expressed in this article are solely those of the authors and do not necessarily represent those of their affiliated organizations, or those of the publisher, the editors and the reviewers. Any product that may be evaluated in this article, or claim that may be made by its manufacturer, is not guaranteed or endorsed by the publisher.
