# Supplementary material for: Prediction of subjective cognitive decline after corpus callosum infarction by an interpretable machine learning-derived early warning strategy
Source: Front Neurol. 2023 Jun 9;14:1123607. doi: 10.3389/fneur.2023.1123607 (PMC10321713; doi:10.3389/fneur.2023.1123607)
Supplement: Supplementary file 1 [file Data_Sheet_1.pdf]

SUPPLEMENTAL MATERIAL

Prediction of Subjective Cognitive Decline after Corpus Callosum Infarction by an Interpretable Machine Learning-Derived Early Warning Strategy.

Supplementary Table

Characteristics	NC (n=103)	SCD (n=110)	P value
Age (years), median [IQR]	61 [51,68]	63 [58,71]	0.012
Female, n (%)	25(24.3)	50(45.5)	0.001
Hypertension, n (%)	67(65.0)	83(75.5)	0.096
Diabetes mellitus, n (%)	40(38.8)	46(41.8)	0.657
Prior stroke or TIA, n (%)	20(19.4)	18(16.4)	0.561
Heart diseases, n (%)	16(15.5)	13(11.8)	0.429
Smoking, n (%)	36(35.0)	28(25.5)	0.131
Alcoholism, n (%)	19(18.4)	15(13.6)	0.338
BMI (kg/m^2), mean (SD)	25.1(4.6)	24.6(2.9)	0.391
Time from onset to hospital (d), mean (SD)	12.3(18.1)	13.5(17.5)	0.642
NIHSS score, mean (SD)	4.2(4.8)	5.2(4.5)	0.119
ALT (U/L), mean (SD)	26.3(22.9)	25.5(30.1)	0.834
LDL (mmol/L), mean (SD)	2.6(1.0)	2.6(0.8)	0.754
HDL (mmol/L), mean (SD)	1.2(0.4)	1.2(0.3)	0.955
Cholestenone (mmol/L), mean (SD)	4.4(1.4)	4.4(1.0)	0.878
Triglyceride (mmol/L), mean (SD)	1.6(1.0)	1.7(1.8)	0.712
Creatine (umol/L), mean (SD)	76.3(25.2)	73.2(24.5)	0.386
Urea (mmol/L), mean (SD)	5.4(2.2)	5.6(2.0)	0.612
Uric acid (umol/L), mean (SD)	119.0(161.3)	80.0(144.2)	0.071
Glu (mmol/L), mean (SD)	7.1(2.8)	7.6(2.7)	0.248
NLR, mean (SD)	6.1(13.7)	4.2(5.7)	0.191
Erythrocyte ($\times 10^{12}/L$), mean (SD)	4.4(0.7)	4.4(0.7)	0.637
Leukocyte ($\times 10^{9}/L$), mean (SD)	7.7(3.3)	7.5(2.7)	0.608
Hemoglobin (g/L), mean (SD)	134.6(22.1)	132.7(20.4)	0.524
Thrombocyte ($\times 10^{9}/L$), mean (SD)	214.6(74.9)	219.9(61.0)	0.579
HCY (umol/L), mean (SD)	1.2(0.4)	1.2(0.3)	0.955
HbA1c (ml/min), mean (SD)	6.9(1.9)	7.4(2.2)	0.160
FIB (g/L), mean (SD)	3.5(1.0)	3.6(0.9)	0.374
D-dimer (ug/ml), mean (SD)	0.7(1.0)	0.7(0.8)	0.736
Pure CC infarction, n (%)	7(6.8)	18(16.4)	0.030

Table S1. Baseline characteristics of patients with acute callosal infarction.

Infarction subregion of CC, n (%)			0.001
Rostrum	0(0)	0(0)	
Genu	24(23.3)	11(10.0)	
Body	7(6.8)	7(6.4)	
Splenium	54(52.4)	48(43.6)	
At least two of the above subregions	18(17.5)	44(44.0)	
Other infarction areas, n (%)			0.117
None	7(6.8)	18(16.4)	
Frontal lobe	20(19.4)	19(17.3)	
Parietal lobe	12(11.7)	7(6.4)	
Temporal lobe	7(6.8)	7(6.4)	
Occipital lobe	16(15.5)	23(20.9)	
Others	41(39.8)	36(32.7)	
Location of angiostenosis, n (%)			0.009
None	19(18.5)	18(16.4)	
ICA	38(36.9)	35(31.8)	
VBA	21(20.4)	14(12.7)	
Both of ICA and VBA	25(24.3)	43(39.1)	
Number of angiostenosis, n (%)			0.009
None	20(19.4)	19(17.3)	
Seldom	27(26.2)	12(10.9)	
Multiple	56(54.4)	79(71.8)	
TOAST subtype, n (%)			0.812
LAA	71(68.9)	83(75.5)	
CE	4(3.9)	4(3.6)	
SAO	12(11.7)	8(7.3)	
ODC	7(6.8)	6(5.5)	
UND	9(8.8)	9(8.2)	
Extracranial carotid plaque, n (%)	58(56.3)	70(63.6)	0.275
3-month mRS score, n (%)	1[0.0, 2.0]	1[1.0, 3.0]	< 0.001
Rehabilitation treatment, n (%)	15(14.6)	15(13.6)	0.846
Regular secondary prevention, n (%)	73(70.9)	80(72.7)	0.764
Recurrent stroke, n (%)	24(23.3)	34(30.9)	0.213

Abbreviation: NC= No-complaint; SCD=subjective cognitive decline; BMI=Body mass index; ALT=Alkaline phosphatase; LDL= Low-density lipoprotein; HDL= High-density lipoprotein; NLR=Neutrophil to lymphocyte ratio; Glu= Glucose; HCY=Homocysteine; HbA1c = Glycosylated hemoglobin; FIB= Fibrinogen; NIHSS= NIH stroke score; CC=Corpus callosum; ICA=Internal carotid artery; VBA=Vertebral basilar artery; LAA=Large-artery atherosclerosis;

CE=Cardiac embolism; SAO=Small-artery occlusion; ODC=Stroke of other determined cause; UND=Stroke of undetermined cause; mRS=Modified Rankin scale.

Supplementary Figure

To investigate for predictive factors of cognitive dysfunction among 45 variables, least absolute shrinkage and selection operator (LASSO) was utilized. Finally, a total of nine features with non-zero coefficients were identified by LASSO regression (5:1 ratio; **Figure S1**), which includes age, female, HCY, NLR, 3-month mRS score, infarction subregions of CC, pure CC infarction, location of angiostenosis, and number of angiostenosis.

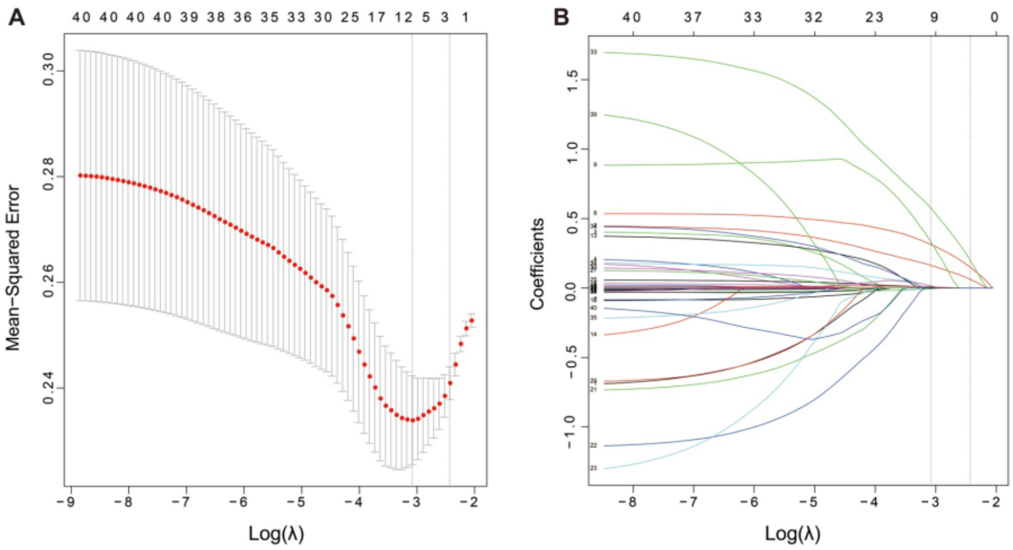

Figure S1. The result of predictive factors selected by LASSO regressive model. **(A)** Partial likelihood deviation map. Based on collinearity minimization, minimum mean square error of λ is 0.046 according to 10-fold cross-validation. **(B)** Trend graph of LASSO coefficients. The vertical line was drawn at the value selected using 10-fold cross-validation, where optimal λ value corresponded to nine non-zero coefficients.
